# Mr. Charles Pearson on the Treatment of Criminal Lunatics

**Published:** 1848-01-01

**Authors:** Charles Pearson, Forbes Winslow

**Affiliations:** Sussex House, Hammersmith


### PROPOSED LEGISLATIVE ENACTMENT IN REFERENCE TO CRIMINAL LUNATICS.

We have been favoured with the following letter from the Honourable Member for Lambeth, Mr. Charles Pearson. It did not arrive until the Journal was made up; but as it refers to a matter of great importance connected with a proposed legislative enactment regarding criminal lunatics, we stop the press for its insertion:—

*To the Editor of the Journal of Psychological Medicine.*

Clapham Common, 21st December, 1847.

Dear Sir,—I am obliged by your letter of the 14th instant, and have much pleasure in complying with your request. The bill it is my intention to introduce into parliament, respecting persons acquitted on the ground of insanity, will principally aim at the rectification of some defects in the existing laws in relation to their disposal after acquittal. The defects I refer to are chiefly of a technical nature in reference to my profession, and would have no interest with the readers of your intended Journal. There is, however, one part of my proposed bill which may fairly give rise to a diversity of opinion amongst the classes to whom your publication will principally address itself, and I shall be most happy to receive the benefit of their and your strictures upon my views. I am of opinion that it would be just and proper to empower the government authorities to whom the custody of criminal lunatics is committed, to exact from the healthy and able-bodied a certain amount of labour, suited to their age, strength, and previous state and condition.

Why should M'Naughten or Oxford be maintained in idleness for the remainder of their days? They are perfectly competent to maintain themselves, and ought, in my judgment, to be required to do so.

I am prepared to encounter the charge of inconsistent legislation, in purposing the introduction of a measure to give effect to this opinion. The law, it will be said, exempts a lunatic from the punishment which belongs to a particular act, upon the express ground that it was done when, by reason of unsoundness of mind, the individual committing it did not know right from wrong, and could not consequently commit a *crime;* and it will be charged as inconsistent to subject such a person to confinement and labour as a consequence of his acquittal, when confinement and labour would probably have been the punishment awarded to the commission of the act if the accused had been of sound mind and criminally responsible for his conduct.

Although I have heard and read much evidence upon this subject, I do not pretend to comprehend the subtleties in which the scientific indulge in this very abstruse matter.

For aught I know to the contrary, it may be true that in a recent case, the attempt to murder his creditor was, as stated by a medical witness, the "crisis" of a "mind off its balance," which, being passed, restored the prisoner to immediate and continued sanity. It may be, as a learned doctor stated, that the youth who poisoned his grandfather was in an "incipient state of insanity, which is likely to develope itself more in the derangement of conduct than the confusion of intellect;" it may be true that habits of waywardness, lying, and obstinacy are tokens of madness: but it appears to me to be the madness of wickedness, and that the best way to "minister to a mind diseased" after this fashion, is to visit its possessor with some salutary discipline.

There are three classes of persons who commit acts for which insanity is the plea, upon each of which I think the prospect and the reality of constant employment, as well as perpetual confinement, would beneficially operate. These classes are:—

1st. Persons who simulate insanity, as a cloak for crime. It is quite clear that this class, though acquitted on the ground of supposed insanity, ought to be made to work. I have met with several instances where persons who have, after their acquittal, avowed that they simulated insanity to escape from punishment, and have yet been kept in idleness and luxury for years at the expense of the nation.

2nd. Persons who, though monomaniacs, have sufficient power, where the motive is strong enough, to control their actions, and to subdue their monomaniacal impulses. History, ancient and modern, affords many striking evidences of this condition of the human mind, and every day's experience confirms the fact of its existence. To such persons, the dread of constant labour and perpetual confinement would supply a powerful motive to check the first tendencies to the aberration of mind and will, which, if unrestrained, may become too powerful for self-control.

3rd. Persons who, from want of mental discipline, co-operating with physical causes, act under impulses they cannot control. If they are possessed of health and strength, there can be no reason, as it appears to me, why they should not be required to maintain themselves. It is, I presume, a well-established fact, that nothing tends so much to the restoration of the mind to its proper tone as healthful labour and continual occupation.

I think there is so much false feeling for criminals, and so much effeminate sentimentality prevailing amongst us, that it is highly probable I shall fail in proving to the satisfaction of the legislature the reasonableness of this measure. I intend, however, to make the attempt; and if you think proper to insert this letter in your forthcoming interesting work, I shall be obliged to your readers for any communications, public or private, which they may think it requires.

I am, dear Sir, yours truly,

Charles Pearson.

To Dr. Forbes Winslow,

Sussex House, Hammersmith.

---

